# Does the routine use of global coronary heart disease risk scores translate into clinical benefits or harms? A systematic review of the literature

**DOI:** 10.1186/1472-6963-8-60

**Published:** 2008-03-20

**Authors:** Stacey L Sheridan, Eric Crespo

**Affiliations:** 1 Division of General Medicine and Clinical Epidemiology, University of North Carolina, Chapel Hill, NC, USA; 2 Division of Cardiology, University of Vermont, Burlington, VT, USA

## Abstract

**Background:**

Guidelines now recommend routine assessment of global coronary heart disease (CHD) risk scores. We performed a systematic review to assess whether global CHD risk scores result in clinical benefits or harms.

**Methods:**

We searched MEDLINE (1966 through June 13, 2007) for articles relevant to our review. Using predefined inclusion and exclusion criteria, we included studies of any design that provided physicians with global risk scores or allowed them to calculate scores themselves, and then measured clinical benefits and/or harms. Two reviewers reviewed potentially relevant studies for inclusion and resolved disagreement by consensus. Data from each article was then abstracted into an evidence table by one reviewer and the quality of evidence was assessed independently by two reviewers.

**Results:**

11 studies met criteria for inclusion in our review. Six studies addressed clinical benefits and 5 addressed clinical harms. Six studies were rated as "fair" quality and the others were deemed "methodologically limited". Two fair quality studies showed that physician knowledge of global CHD risk is associated with increased prescription of cardiovascular drugs in high risk (but not all) patients. Two additional fair quality studies showed no effect on their primary outcomes, but one was underpowered and the other focused on prescribing of lifestyle changes, rather than drugs whose prescribing might be expected to be targeted by risk level. One of these aforementioned studies showed improved blood pressure in high-risk patients, but no improvement in the proportion of patients at high risk, perhaps due to the high proportion of participants with baseline risks significantly exceeding the risk threshold. Two fair quality studies found no evidence of harm from patient knowledge of global risk scores when they were accompanied by counseling, and optional or scheduled follow-up. Other studies were too methodologically limited to draw conclusions.

**Conclusion:**

Our review provides preliminary evidence that physicians' knowledge of global CHD risk scores may translate into modestly increased prescribing of cardiovascular drugs and modest short-term reductions in CHD risk factors without clinical harm. Whether these results are replicable, and translate across other practice settings or into improved long-term CHD outcomes remains to be seen.

## Background

Despite numerous guidelines and abundant evidence regarding the efficacy of interventions to prevent cardiovascular disease (CVD), the majority of people with CVD risk factors do not have them under adequate control [1-5]. One contributing factor is that many clinicians do not accurately estimate a patient's risk of CVD [6-10]. This may lead to under use of effective therapies and, in some cases, excess harms.

Several major guidelines now advocate routine assessment of cardiovascular risk using coronary heart disease (CHD) risk scores as a means to aid clinicians in decision-making [11-13]. The major benefits in using global CHD risk scores are improved prediction of CHD outcomes [14] and improved physician knowledge of a patient's actual risk. These may in turn result in earlier identification of high-risk patients who require immediate attention, more appropriate allocation of therapies to those most likely to benefit, and improved intermediate and long term outcomes for patients [15,16]. To the extent that the risk information is communicated to patients, global CHD risk scores may also improve patient understanding of their risk for CVD and the rationale for any proposed treatments, and patient motivation to adhere to prescribed risk-reducing interventions [13].

Despite clear-cut recommendations for the use of global CHD risk scores, relatively little is known about whether use of such assessments actually translates into improved clinical outcomes [17]. In particular, little is known about how knowledge of a patient's calculated 10-year CHD risk affects physicians' actions (i.e. prescribing or adherence to guidelines) or whether these actions translate into improved outcomes for patients through improved acceptance of or adherence to CHD risk-reducing therapies. We undertook this systematic review to summarize available evidence about the effects of physician knowledge of global CHD risk.

## Methods

### Questions to be reviewed

The primary question to be addressed by this systematic review is: Does physician knowledge of a global CHD risk scores (as opposed to either simple risk factor counting or no formal assessment of risk) translate into clinical benefits? For the purposes of this review, we defined clinical benefits as either (1) improved physician adherence with evidence-based guidelines for the primary prevention of CVD, (2) increased appropriate prescribing of risk-modifying therapies, (3) increased patient acceptance of or adherence to therapies targeted at the primary prevention of CVD, (4) improved control of patient CVD risk factors (e.g. blood pressure, cholesterol), or (5) a reduction in CVD events.

Our secondary question was whether there are any harms (from the patient perspective) associated with screening using global risk scores. Since harms can be difficult to predict, we chose to broadly define clinical harms as any adverse physical or psychosocial outcome that correlated with screening.

### Search strategy

We searched MEDLINE (1966 – June 13, 2007) using MeSH terms in two distinct search strategies (see Additional file 1). Each search was limited to studies in humans and to the English language literature. To augment our findings, we additionally performed related articles searches of included articles in MEDLINE and hand-searched the bibliographies of included articles and our files looking for additional articles on the effects of CHD risk calculation on clinical outcomes.

### Study inclusion criteria

In our search for articles about the benefits of physician knowledge of global CHD risk scores, we included studies of any design that met the following criteria: (1) study population consisted of adults (>18 years old) with no prior history of CVD; (2) global CHD risk calculation was specified as the primary study intervention; (3) there was clear documentation of the calculation of a global CHD risk score by a physician or other health care provider as part of an individual patient encounter (Note: questionnaire-based studies were considered acceptable as long as they were designed to simulate clinical encounters with patients); and (4) one or more of the following endpoints was used: (i) rates of prescribing for aspirin, anti-hypertensive medication, lipid-lowering medication, smoking cessation therapies, or diet and exercise; (ii) physician compliance with guidelines for CVD prevention; (iii) patient adherence with therapy; (iv) change in patient blood pressure, cholesterol levels, aspirin use, smoking cessation, diet or exercise; or (v) rate of CVD events (defined here as new onset stroke/transient ischemic attack, myocardial infarction, acute coronary syndrome, stable angina, peripheral vascular disease, carotid artery disease, or cardiac death).

In our search for articles about the potential harms associated with physician knowledge of global CHD risk scores, we included studies that met the following criteria: (1) study population consisted of adults (>18 years old) with no prior history of CVD; (2) assessment of the adverse effect of global CHD risk calculation was specified as the primary study goal; and (3) one or more of the following patient-specific endpoints was used: (i) general health and wellbeing, (ii) anxiety or worry, (iii) depression, or (iv) motivation to lower CHD risk.

In both searches, we considered risk calculation using Framingham-derived estimates preferable, however other scoring systems were acceptable as long as they presented risk in a comparable fashion (i.e. as an absolute risk estimate or in terms of risk categories – low, intermediate, high). Additionally, in order to be included, studies had to provide enough information so that it was possible to determine the method of global risk calculation as well as the manner in which the risk assessment was used in the clinical encounter.

### Study exclusion criteria

In our search for articles about the benefits of physician knowledge of global CHD risk scores, we excluded studies for the following reasons: (1) studies were unrelated to global CHD risk calculation; (2) they calculated the risk scores for the secondary prevention of CHD risk; (3) they answered the wrong question about CHD risk scores (e.g. they were related to development and validation of risk scores; the conceptual understanding of risk scores; the accuracy of risk perception; the accuracy of systems to calculate CHD risk; or the acceptability of decision aids including CHD risk); (4) they used risk scores for the wrong use (e.g. as part of the eligibility criteria or endpoints of a study, but not as information for physicians); (5) they were about the benefits of CHD risk scores, but did not have a quantitative experimental design (e.g. reviews, editorials, qualitative studies, methods papers); (6) they used a non-controlled experimental design (e.g. pre-post designs); (7) they focused on the effects of patient knowledge of CHD risk scores; or (8) they administered global risk as part of a mixed intervention without the ability to determine the independent effect of the risk score.

In our search for articles about the potential harms associated with physician knowledge of global CHD risk scores, we excluded articles because (1) they were unrelated to the harms of CHD risk disclosure; (2) they were related to CHD risk disclosure, but were not about disclosing *global *CHD risk; (3) they answered the wrong question (e.g. the effects of disclosure in secondary prevention); or (4) they didn't have an experimental design.

### Data extraction strategy

Two reviewers (EC, SS) independently reviewed titles, abstracts, and, if necessary, full articles to determine inclusion. Disagreements were resolved by discussion. Once consensus about article inclusion was achieved, one reviewer (EC or SS) abstracted information about study features into tables for analysis and two reviewers independently assessed study quality.

### Assessment of study quality

We assessed study quality using criteria adapted from the U.S. Preventive Services Task Force [18]. Under these criteria both research design and internal validity are taken into account when assessing the quality of an individual study.

The study grade for internal validity was based on fulfillment of the following criteria: (1) creation and maintenance of comparable study groups; (2) valid and reliable measurement that is applied equally to both study groups; (3) provision of a clear definition of the intervention; and (4) performance of appropriate statistical analysis, including appropriate control for confounding and accounting for cluster randomized design if necessary. For each study, we graded each criterion as good, fair, or poor. We then converted our quality ratings for each item into numeric values (0 = *poor*, 1 = *fair*, and 2 = *good*) and created a composite rating for each study. We gave each item equal weight and excluded items we judged to be not applicable based on study design. We totaled and averaged scores for each evaluator and then averaged scores from the two evaluators, giving a final score ranging from 0 to 2. We based our final quality grades on averaged scores according to the following scale: a mean score ≥ 1.5 was considered good quality; a mean score of 1.0 to 1.49 was considered fair quality; and a mean score < 1.0 was considered to denote a study with significant methodological limitations.

In order to account for study information which may have been collected but which was not included in the published article (due to space limitations, etc), we requested further information from the contact authors via email when we had specific questions about information that was missing. We sent reminders to contacted authors who did not respond within one month after our initial contact.

## Results

### Search results

**Figure 1:**
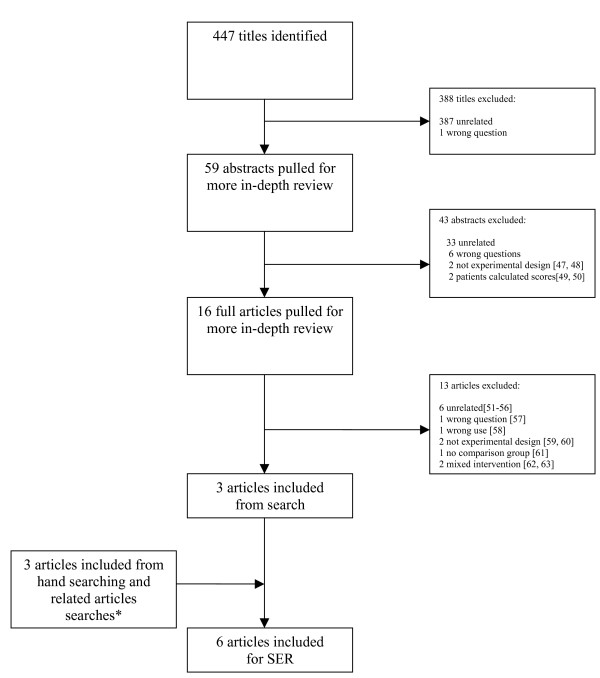
**Literature search results for benefits of physician knowledge of global CHD risk scores**. * Note: Several articles that may be of interest to readers were not included from related articles searches because they: did not have an experimental design [64-69], had no comparison group [70-79], had patients rather than physicians calculate risk scores [80-82], or had mixed interventions [83-89].

**Figure 2:**
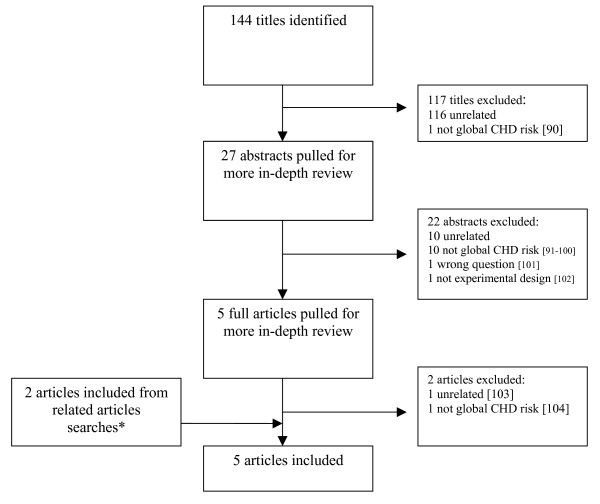
**Literature search results for harms of physician knowledge of global CHD risk scores**. *Note: Two articles that may be of interest to readers were not included from related articles searches because they reported on the harms of CVD screening (but not presentation of global CHD risk) [105, 106].

We summarize the results of our two literature searches in Figures 1 and 2. In our search for articles about clinical benefits, we identified 6 studies for inclusion in our review. We identified three through our primary literature search, one through hand searching, and two through related articles searches.

In our search for articles about the harms, we identified 5 articles for inclusion. We identified three articles through our primary search and two through related articles searches.

### Study characteristics

A total of eleven studies were included in our review: six addressed whether physician knowledge of a global risk score translates into improved clinical outcomes (see Additional file 2) [19-24]; five addressed whether global risk scores are associated with harm (see Additional file 3) [25-29]. All included studies were published after 1995.

Of the six studies that addressed clinical benefit, three were cluster randomized controlled trials, two were traditional randomized controlled trials, and one was a cross-sectional study. Five took place in actual clinical settings [20-24], whereas a sixth study queried physicians using hypothetical patient scenarios[19]. Of those in actual clinical settings, four [20,21,23,24] took place in a general practice population and one [22] was conducted in a diabetes referral clinic. All six studies used some form of a Framingham-derived risk score [19-24]. In three studies, risk scores were provided to the physician [21-23]. The number of patients and, more importantly, physicians included in each study varied widely, with the study by Hall being the smallest (6 physicians, 323 patients) and the study by Lowensteyn being the largest (253 physicians, 958 patients). As can be seen in Additional file 2, the primary endpoints of the six studies also varied widely. No studies addressed actual CVD event rates or the effect of risk scores on patient adherence.

Of the five studies that addressed potential harms, two were randomized controlled trials, two were cohort studies, and one was a nested cross-sectional study. All took place in general practice populations (Additional file 3). Three studies used risk scores derived from epidemiologic databases other than Framingham (e.g the Northwick Park Heart Study [26], the British Regional Heart Study and Dundee risk score [25], and the Norwegian Infarction Score [29]). Two studies used a risk score that was a composite of data from Framingham, the Pooling Project, and the NHBLI working group on Arteriosclerosis [27,28,30]. In all studies, the risk scores were presented in a categorical format (low, intermediate, high) similar to Framingham scores.

### Study quality

Despite obtaining additional information from 10 of 11 authors [19-23,25-29], study quality ratings ranged from "methodologically limited" to "fair" (see Additional file 4). Across all of the studies a major limitation in determining both the internal validity of each study was a lack of detailed information about the study procedures and the baseline characteristics of both the physicians and the patients. This created difficulty in assessing the adequacy of randomization, selection bias, and confounding.

### The benefits of risk calculation

#### Changes in physicians' prescribing habits

Four fair quality studies [20,22-24] examined the effects of CHD risk calculation on physicians' prescribing habits. Although only the Hall, Jacobsen, and van Steenkiste studies considered prescribing habits a primary outcome, the study by Montgomery provides important evidence due to its larger sample of physicians and its appropriate accounting for the effects of clustering.

In the study by Hall and colleagues, six diabetologists saw 323 diabetic patients without known CHD who were pseudo-randomized (e.g. alternately allocated) to an experimental group (5-year CHD risk scores (New Zealand risk score) placed on the front of the chart prior to visit) or to a usual care group (no risk information provided). Outcomes measured post-visit included a documented change in treatment of diabetes, prescription rates for lipid lowering or antihypertensive drugs, and referrals to a dietician. Overall, documentation of a risk score did not have an effect on physician prescribing habits; however, in an a priori subgroup analysis of high risk patients (5-year CHD risk > 20%; encompassing 52% of the patients in the study), there was a trend toward more prescribing of a lipid lowering and anti-hypertensive drugs when risk was documented on the chart (23% vs. 10% for antihypertensive agents and 20% vs. 9% for lipid-lowering agents). Additionally, after adjusting for the level of risk, the difference in prescribing of lipid lowering and anti-hypertensive drugs between the intervention and control groups was significant (Mantel Hansel chi square, p < 0.02). There was no difference in prescribing of diabetes treatment or referrals to dieticians.

The study by Montgomery and colleagues supports these findings. In this study, 27 practices (including 74 physicians and 11 nurses) were randomized to receive one of three interventions: (1) a CHD risk chart (New Zealand risk chart), (2) a CHD risk chart plus a computer-based clinical decision support system that calculated risk using New Zealand chart principles, or (3) usual care. Outcomes, including the proportion of patients at high risk, physician prescribing habits, and blood pressure, were measured at 12-month follow-up on 614 patients, 86% of whom were at high risk (mean 5 year CHD risk 18.5%). After adjustment for practice site (n = 27) and for baseline CHD risk, a similar proportion of patients in all three groups were still at high risk. Physicians in the chart only group, however, had two times the odds of prescribing cardiovascular drugs as physicians in the other two groups (p < 0.01), and the patients in this group had significantly lower systolic blood pressure at 12 months (-4.6 mmHg; 95% CI -8.4 to -0.8).

The study by Jacobsen reached different conclusions, although results should be interpreted with caution due to the lack of adequate power for primary and secondary analyses and the unique physician population in this study. In this study, 164 medical residents at a university-based general medicine clinic saw 368 patients who were randomized to an experimental group (10-year CHD risk scores plus a check list for physicians to indicate treatment plans, placed on the front of patients' charts prior to visits) or a control group (educational form conveying primary prevention targets, placed on the front of patients' charts prior to visits). The primary and secondary outcomes were physicians' prescription of statin medications for lipid lowering to high (10-year risk > 20%; encompassing 18% of the patients in the study) and moderate risk patients (10-year CHD risk 10–19%; encompassing 35% of patients in the study). Other outcomes included physicians' prescription of statins, hypertension medications, aspirin, or lifestyle changes to patients at all levels of risk. Investigators found no difference in prescription of statin medications to high (+2%, p = 0.86) or moderate risk patients (+10%, p = 0.18) by intervention assignment. Changes in other outcomes were also non-significant between groups, except for increased referrals (not counseling) for smoking cessation (+13%, p < 0.01) and less dietary counseling (-10%, p 0.01; but not referrals) among the intervention group, which may have resulted from multiple testing.

The study by van Steenkiste and colleagues also failed to find effects on prescribing, although results should be interpreted in the context of their lower risk sample, their target for prescribing (which was lifestyle changes that would be appropriate for all patients regardless of risk level), and moderate loss to follow-up, which diminished the chance for physicians to make decisions based on global CHD risk. In this study, 39 general practices (including 45 interested physicians) were randomized to a 3-part intervention (physician education, a paper-based decision support tool to help physicians calculate global CVD risk and explain it to their patients, and two scheduled medical consultations to encourage risk reduction) or a control group (educational materials on the Dutch National Cholesterol Guidelines). Physician level outcomes included appropriate ordering of cholesterol testing for potentially high risk patients (those with missing data for risk calculation, encompassing 59% of the study population), and appropriate dietary and smoking advice for all patients (including 19% who were high risk by Dutch National Guidelines with CVD risk > 20% at 40 years, > 60% at 60 years, or diabetes). Patient level outcomes included changes in risk perception, smoking, and physical activity (but not diet) at 26 weeks. After adjustment for clustering, there were no appreciable differences in physicians' dietary or smoking advice. There were also no changes in patients' risk perception or self-reported smoking. Interestingly, there were differences in patients' self-reported physical activity at 26 weeks (+11%, p < 0.05); however, it is not clear whether these were related to the intervention, unaccounted for confounding with failed randomization, or multiple testing.

#### Physician compliance with guidelines

One methodologically limited cross-sectional study by Ramachandran [19] examined the effects of CHD risk calculation on physician compliance with guidelines. In this study, 68 randomly-selected general practitioners (GPs) in the UK completed a postal survey in which they indicated the need for lipid treatment in each of 20 case scenarios (mean 10-year CHD risk 28.9%). The primary outcome was the proportion of correct responses based on the UK lipid guidelines, which, at that time, recommended lipid-lowering therapy for all patients with a 10-year CHD risk > 30%. GPs were reminded of this guideline in the cover letter that accompanied the questionnaire. Unfortunately, design limitations, most notably the small sample size and the response rate of only 30.5%, seriously limit the study's primary conclusion that there was no difference in the appropriateness of decision-making between GPs who made recommendations after calculating CHD risk (N = 52) and those who made recommendations without calculating risk (N = 26, P = 0.21).

#### Changes in CHD risk factors and global CHD risk

Three studies examined the effects of CHD risk calculation on changes in risk factors and global CHD risk.

Two of these studies have been discussed in detail above. As already noted, a fair quality cluster randomized trial by Montgomery [20], showed that risk calculation using a chart-based risk calculation system was associated with lower mean systolic blood pressure at 12 months compared to use of no risk calculation system in a high risk population (86% of individuals at high risk). Risk calculation resulted in no difference in the proportion of individuals at high risk (CHD risk > 10%), however, perhaps because of the high proportion of patients whose baseline risk was significantly above the 10% threshold. A fair quality cluster randomized trial by van Steenkiste [24], on the other hand, showed mixed effects on CHD risk factors (increased self reported physical activity (+11%, p < 0.05) and unchanged self-reported smoking). This study, however, targeted lifestyle changes that would be appropriate for all patients regardless of risk level, and was conducted in a lower risk sample with moderate loss to follow-up (diminishing the chance of physicians to make decisions based on global CHD risk) and unaccounted for confounding.

A third study by Lowensteyn and colleagues [21] found reductions in some (but not all) risk factors and in CHD risk; however interpretation of these results is even more difficult due to the study's methodological limitations. In this study, 253 Canadian physicians recruited from a continuing medical education meeting on cardiovascular risk assessment were randomized to receive either a computerized risk report on their patients within 10 working days of their patient's clinic visit (profile group) or no report (control group). Two-week follow-up visits were scheduled for all patients in the profile group so that CHD risk could be communicated to patients; subsequent follow-up was at the discretion of the physician and patient. Risk factor information was collected on control patients, but no risk profile was provided until after the study period. The authors hypothesized that assessing CHD risk at baseline would encourage the 63% of patients who were at high-risk in this study to be seen for follow-up and that this would be associated with enhanced control of CHD risk factors. Although the likelihood of follow-up for high versus low risk persons was higher in the profile group than the control group (difference 0.46, 95% CI 0.08 to 0.87), the differential follow-up rates at 3 months between the two study arms (50% profile group; 25% control), the low physician response rate, and the use of a convenience sample of patients, all combine to hamper interpretation of the secondary outcome of risk factor change, which suggested that patients in the profile group had significantly greater reductions in lipid values and calculated 8-yr CHD risk, but not in blood pressure or smoking (see Additional file 2).

#### Changes in CHD events

We found no studies that addressed the effect of physician knowledge of global CHD risk scores on changes in actual CHD events.

### The harms of CHD risk calculation

We found five studies examining the effects of patient knowledge of CHD risk calculation on psychological outcomes and health status (see Additional file 3). Quality ranged from fair [27,28] to methodologically limited [25,26,29].

#### Psychological outcomes

Four studies examined the effects of patient knowledge of global CHD risk scores on psychological outcomes [26-29]. Three of these studies examined effects on the General Health Questionnaire (GHQ-12 or 28). The GHQ is a self-administered questionnaire that was designed and validated to detect psychiatric disorders in non-psychiatric settings [31,32], but has been widely used to capture the harms of screening [33,34] given the face validity of questions, which focus on anxiety, depression, hypochondria, and social dysfunction. The fourth study examined effects on overall satisfaction with life, measured by a single item Likert scale with moderate correlation with the GHQ.

In a prospective cohort study by Connelly [26], 5772 men in 9 practices underwent CHD screening and were informed of their level of CHD risk (low or higher than average in 6 practices; and low, moderate, or high in 3 practices) via mailed communication. Men categorized as "high" or "higher than average" risk (18.5%) were scheduled for a follow-up appointment to discuss their results and were invited to participate in a clinical trial. Men categorized as "moderate" risk received general advice about their risk factors with their mailing but no scheduled follow-up visit. Men categorized as "low" risk simply received the mailing stating they were at low risk. Psychological symptoms were assessed using the GHQ-28 at the baseline, after the mailing, and at 3-month follow-up. Results indicated that men who were labeled as being at either "high," "higher than average," or "low" risk showed a decrease in their psychological symptoms after labeling, but men labeled as "moderate" risk had an increase in psychological symptoms (see Additional file 3). The lack of a scheduled follow-up appointment in the moderate risk group may explain this result, although the lack of reporting of baseline characteristics and substantial patient attrition (~35%) make interpretation difficult.

Two fair quality studies by Christensen [27,28] support the hypothesis that counseling and scheduled follow-up may mitigate any potential adverse effects from knowledge of CHD risk scores. In the first study [27], 52% of 2452 men randomly sampled from 2 municipalities in one county in Denmark attended a health screening and had their CHD risk calculated. Patients at all risk levels had immediate counseling and those designated to be at increased or high risk (n = 164) were scheduled a second examination. All men at increased or high risk completed a GHQ-12 at baseline and 6 months, as did a random sample of men at low or moderate risk (n = 188). Investigators found no differences in GHQ scores at 6 months among men designated to have these different risk levels (change in GHQ score -0.20, p 0.8). In the second study by Christensen [28], a random sample of 1507 patients from all 9 practices in one county in Denmark were randomly assigned to receive one of two screening interventions (e.g. health screening with written notification of their global CHD risk from their provider plus either 1) optional or 2) scheduled yearly follow-up) or no screening. Men who received scheduled follow-up helped set the agenda for their visit and were invited to set a maximum of three health-related lifestyle goals for the following year, which were confirmed in writing [35]. Outcomes were measured at 12 months and five years and showed no differences between the intervention groups or the intervention and control groups in changes in the GHQ-12.

One final nested cross-sectional study by Meland [29] also argues that scheduled follow-up may mitigate against any potential adverse effects from knowledge of CHD risk scores, although results must be interpreted with caution given the high potential for confounding. In this study, investigators compared the overall satisfaction with life of 115 high risk men enrolled in a randomized trial of heart disease prevention (including every 3 month follow-up) with the overall satisfaction with life of a random sample of low risk men (n = 92, comprising 61% of those sent a postal query) who were excluded from participation in the trial. Baseline characteristics of participants were not provided, but significant differences would be expected based on risk alone. Satisfaction with life was measured with a single question at screening and inclusion, which showed moderate correlation to the GHQ-28, and was no different in men who were labeled as high risk and enrolled in the trial and those who were low risk and precluded from enrollment (between group difference +0.1 on a 7-pt Likert scale, p0.9).

#### Perceived health status

A single methodologically limited randomized controlled trial by Marteau [25] examined the effects of patient knowledge of CHD risk scores on perceived general health status. In this study 3000 couples were randomized to screening, including CHD risk calculation, or no screening. All patients who had a CHD risk calculated were counseled on ways to reduce CHD risk, and they were offered follow-up at a frequency commensurate with their level of risk (i.e. more frequent follow-up for higher risk patients). Outcomes included perceptions of health, the risk of suffering a heart attack, and the ability to reduce CHD risk at 1 year. Results suggest that participation in a screening program including calculation of a CHD risk score and appropriate counseling was not associated with adverse concerns about health, although subjects who were screened did have a sense of less ability to lower their risk. Unfortunately, limited data about the baseline characteristics of participants (even in background studies) and patient follow-up with the possibility for confounding limit conclusions from these findings.

#### Motivation

A single methodologically limited study by Marteau [25] examined the effects of patient knowledge of risk scores on motivation. This study was discussed in detail above. As already noted, this study showed that subjects who participated in a screening program that included calculation of a CHD risk score has a reduced sense that they could lower their risk (-6.4%, p < 0.001). Interestingly, this lower self efficacy was associated with greater risk reduction, suggesting that the lower self efficacy may have resulted from activated patients exhausting their options for risk reduction or reaching personally acceptable levels of risk. Unfortunately, authors did not have data to examine possible explanations further and methodological issues hamper interpretation.

## Discussion

Clinical guidelines have suggested that calculation of global CHD risk is a useful addition to the clinician's armamentarium to reduce the burden of CHD in the population. However, we found surprisingly little evidence that physician knowledge of global CHD risk currently translates into improved clinical outcomes. We found two fair quality studies that showed that physician knowledge of global CHD risk is associated with increased prescription of cardiovascular drugs in high risk (but not all) patients. Two additional fair quality studies showed no effect on their primary outcomes, but one was underpowered and the other focused on prescribing of lifestyle changes, rather than drugs whose prescribing might be expected to be targeted by risk level. One of these aforementioned studies showed improved blood pressure in high-risk patients, but no improvement in the proportion of patients at high risk, perhaps due to the high proportion of participants with baseline risks significantly exceeding the risk threshold. Other studies were too methodologically limited to draw conclusions regarding the effect of physician knowledge of global CHD risk on beneficial intermediate outcomes and no studies addressed the effect of physician risk calculation on actual CHD outcomes. Importantly, five studies (including two of fair quality) examined harms and found no evidence of harm from patient knowledge of global risk scores when they were accompanied by counseling, or optional or scheduled follow-up.

The finding that physicians increased their prescribing of cardiovascular drugs in high risk (but not all) patients suggests that global CHD risk scores may be having their intended effect: increasing allocation of therapies to those most likely to benefit. Current guidelines recommend aspirin and lipid lowering therapy for high risk patients; and, evidence supports a similar approach for hypertension therapy [11,36]. By contrast, diabetes guidelines are *not *risk-based because diabetes appears to be a CHD risk equivalent [37]. Thus, the lack of effect of the intervention on diabetes treatment in the Hall study could be seen, not as a failure of global risk scores, but as a marker of good clinical care. Similar reasoning might be applied to the lack of diet, exercise, and smoking counseling in the studies by Hall, Jacobsen, and van Steenkiste [22-24]: these are indicated regardless of CHD risk due to their efficacy in preventing the development of either CHD risk factors or other illness.

Despite increased prescribing of blood pressure and lipid lowering drugs, the lack of effect of physician knowledge of global risk scores on CHD risk in the Montgomery study raises concerns about whether global risk scores will ultimately translate into improved clinical outcomes. Although the lack of effect may be due to measurement of CHD risk as a categorical variable (> 10% or not), it may also be due to a host of patient factors that are intermediary to improved clinical outcomes. To effect improved outcomes, global risk scores must be communicated to patients, increase their perceived risk, and increase their acceptance and long-term adherence to prescribed therapy. Almost none of these intermediate steps were measured in the studies in this review. Several studies have examined the effect of giving global risk information directly to patients (rather than physicians), however [38-40]. In aggregate, these studies suggest that giving patients global risk information in combination with individualized counseling and/or detailed group education (but not alone) may result in reductions in CHD risk factors (e.g. poor diet, physical inactivity, obesity, and smoking) and CHD risk. Unfortunately, none of these studies examined the impact of global risk information on medication use, although one showed higher patient intent to use medication when compared with usual care [40].

To adequately judge the clinical impact of global risk scores, more work is needed. Studies need to define a conceptual framework for the impact of global risk calculation and more explicitly measure the potential mediators of improved outcomes. This will allow a more accurate assessment of whether risk scores have the potential to effect changes in clinical outcomes. Risk is a difficult concept, even for highly educated people [41]. Thus, future studies need to be designed to identify whether physicians and patients appropriately interpret and use risk scores or whether changes in outcomes are due to the presentation of ancillary information, particularly about risk factors themselves.

Future studies also need to determine what happens when physicians are asked to calculate risk (as they would in clinical practice) rather than just interpret its meaning. Montgomery's finding that patient risk factor profiles improved when physicians were randomized to use a risk chart, but not a computer-based intervention, raises questions about physicians' willingness to expend effort in calculating global CHD risk. Through personal communication, we learned that physicians in Montgomery's study had computers available in all exam rooms, but neither the actual use of the computer-based intervention, nor the additional time and effort needed to use it were measured. Future studies of risk calculation interventions should measure both the use of the intervention and environmental factors that are known to be barriers to physician guideline adherence [42].

Future studies should additionally take care to address the methodological challenges evident in studies included in our review. Most importantly studies should address the effects of clustering. Clustering can occur in two situations. First, it occurs when groups of individuals are randomized and inferences are made about members of those groups who are nested both within their group and their study assignment. This nesting and the non-random similarity of members within a group, if unaccounted for, increases the likelihood that studies will draw falsely positive conclusions [43,44]. Three cluster randomized studies [20,21,24] in our review demonstrate this situation and appropriately accounted for clustering. Second, in a more subtle, but related, situation individuals are randomized in a traditional randomized trial, but naturally fall within a cluster (i.e patients are seen by a single physician) that leads to similarity in behavior (i.e. individual physician practice pattern leads to similar outcomes among patients) [43,44]. This situation, if unaccounted for, can lead to falsely negative results. This situation occurred in the study by Hall and colleagues [22], who didn't account for clustering. This raises the possibility that there might have been an effect of physician knowledge of global risk scores among all patients (as well as high risk patients) that might have been detected if they had accounted for clustering. This situation also appeared to have occurred in the study by Jacobsen [23], however, authors reported in personal communication that they examined the effects of clustering and they were minimal.

As the aforementioned design and methodological issues are addressed, the clinical and research communities have one additional important task: to define what ultimately constitutes an acceptable measure of success in the use of global risk scores. Although we found a lack of studies examining the effect of global risk scores on actual CHD events, we do not feel that future studies need to measure the outcome of CHD events. Not only is the time and cost necessary for this measure prohibitive, but previous research has clearly established link between reductions in CHD risk factors and CHD events [45,46]. We additionally wonder whether global risk scores must be held to the standard of measuring change in CHD risk factors or actual CHD risk. Although risk scores would ideally improve patient adherence, they may be considered successful enough if they only increase acceptance of appropriate risk reducing therapies.

Observations aside, we must acknowledge the limits of our own review. First, although a systematic literature search was done, there remains the possibility that relevant literature may not have been located. The questions posed by this review lend themselves to a variety of research designs by investigators in different fields, making identification of relevant literature difficult. Additionally, we searched only one database to identify literature and excluded non-English language articles. Second, although we used a quality assessment method that was based on the best available evidence and has been used in other similar reviews, there is the potential for misclassification in quality because this measure has not previously validated. We do not believe that these issues outweigh the overall value of our work.

## Conclusion

Our review provides preliminary evidence that physicians' knowledge of global CHD risk scores may translate into modestly increased prescribing of cardiovascular drugs and modest short term reductions in CHD risk factors. Whether these results are replicable, and translate across other practice settings or into improved long-term CHD outcomes remains to be seen. An important additional area of study is whether risk scores cause unintentional harm; limited available evidence suggests they don't when accompanied by appropriate clinical support.

## Competing interests

The author(s) declare that they have no competing interests.

## Authors' contributions

SLS and EC jointly conceived of the study, participated in its design, abstracted data and assessed its quality, performed interpretation of data, and prepared the manuscript.

## Pre-publication history

The pre-publication history for this paper can be accessed here:



## Supplementary Material

Additional file 1 Literature search strategy, literature search strategy Click here for file

Additional file 2 Characteristics of studies addressing the clinical benefits of global risk scores, table summarizing study characteristics Click here for file

Additional file 3 Characteristics of studies addressing the harms of global risk scores, table summarizing study characteristics Click here for file

Additional File 4 Summary of overall study quality rating, table summarizing study quality ratings Click here for file
