# Supplementary material for: Does the routine use of global coronary heart disease risk scores translate into clinical benefits or harms? A systematic review of the literature
Source: BMC Health Serv Res. 2008 Mar 20;8:60. doi: 10.1186/1472-6963-8-60 (PMC2294118; doi:10.1186/1472-6963-8-60)
Supplement: Additional file 1 — Literature search strategy, literature search strategy [file 1472-6963-8-60-S1.doc]

**Additional file 1: Literature search strategy**

Search strategy (BENEFITS)

- Search strategy 1: "Coronary Disease/prevention and control"
- Search strategy 2: "Decision Making, Computer-Assisted" OR "Decision Making"
- Search strategy 3: "Hyperlipidemia" OR "Hypertension" OR "Smoking" OR "Aspirin"
- Search strategy 4: "Coronary Disease/prevention and control" OR ("Hyperlipidemia" OR "Hypertension" OR "Smoking" OR "Aspirin"), Limits: All Adult: 19+ years, English, Publication Date from 1966 to 2005/06/01, Humans
- Search strategy 5: ("Coronary Disease/prevention and control" OR ("Hyperlipidemia" OR "Hypertension" OR "Smoking" OR "Aspirin")) AND ("Decision Making, Computer-Assisted" OR "Decision Making"), Limits: All Adult: 19+ years, English, Publication Date from 1966 to 2007/06/13, Humans

Search strategy (HARMS)

- Search strategy 1: "Cardiovascular Diseases"
- Search strategy 2: "Mass Screening"
- Search strategy 3: ("Psychology"[MeSH] OR "psychology"[Subheading])
- Search strategy 4: "Mass Screening" AND ("Psychology"[MeSH] OR "psychology"[Subheading])
- Search strategy 5: "Cardiovascular Diseases" AND "Mass Screening" AND ("Psychology"[MeSH] OR "psychology"[Subheading])
- Search Strategy 6: "Cardiovascular Diseases" AND "Mass Screening" AND ("Psychology"[MeSH] OR "psychology"[Subheading]), Limits: All Adult: 19+ years, English, Publication Date from 1966 to 2007/06/13, Humans
