# Supplementary material for: Does the routine use of global coronary heart disease risk scores translate into clinical benefits or harms? A systematic review of the literature
Source: BMC Health Serv Res. 2008 Mar 20;8:60. doi: 10.1186/1472-6963-8-60 (PMC2294118; doi:10.1186/1472-6963-8-60)
Supplement: Additional file 2 — Characteristics of studies addressing the clinical benefits of global risk scores, table summarizing study characteristics [file 1472-6963-8-60-S2.doc]

**Additional file 2: Characteristics of studies addressing the clinical benefits of global risk s**cores

| **Study/**  **Research Objective** | **Design & Setting** | **Sample size** | **Unit**  **Of**  **Random-ization** | **Comparison** | **Duration**  **of Follow-up** | **Endpoints** | **Outcome** | | |
| --- | --- | --- | --- | --- | --- | --- | --- | --- | --- |
|  |  |  |  |  |  |  | **Control** | **Intervention** | **Difference** |
| Hall et al.  2003  *To determine if documentation of a global CHD risk score improves management of risk factors among diabetic patients* | RCT  Diabetes Clinic, UK | 323 patients;  6 physicians | Patient | **Intervention:**  Documentation of CHD risk score on the front of the patient’s chart  **Control:**  No documentation of risk score (implied) | NR | (1) Change in diabetes treatment  (2) Prescription of lipid-lowering or anti-hypertensive drugs  (3) Referral to a dietician | **(1) Diabetes Treatment**  ***All***  36% (95% CI 29 to 45%)  ***High Risk***  35% (95% CI 24 to 47%)  **(2) Lipid Lowering Treatment**  ***All***  9% (95% CI 4 to 14%)  ***High Risk***  9% (95% CI 2 to 15%)  **(2) Antihypertensive Drug Treatment**  ***All***  10% (95% CI 5 to 16%)  ***High Risk***  10% (95% CI 3 to 17%)  **(3) Referral to Dietician**  ***All***  13% (95% CI 7 to 19%)  ***High Risk***  7% (95% CI 1 to 17%) | **(1) Diabetes Treatment**  ***All***  42% (95% CI 34 to 50%)  ***High Risk***  44% (95% CI 35 to 54%)  **(2) Lipid Lowering Treatment**  ***All***  12% (95% CI 7 to 17%)  ***High Risk***  20% (95% CI 12 to 27%)  **2) Antihypertensive Drug Treatment**  ***All***  16% (95% CI 10 to 22%)  ***High Risk***  23% (95% CI 15 to 31%)  (**3) Referral to Dietician**  ***All***  10% (95% CI 6 to 15%)  ***High Risk***  10% (95% CI 5 to 16%) | **(1) Diabetes Treatment**  ***All***  6% (95% CI -5 to 17%)*  ***High Risk***  9% (95% CI -6 to 24%)*  **(2) Lipid Lowering Treatment**  ***All***  3.6% (95% CI -3 to 10% ) *  ***High Risk***  11% (95% CI 1 to 22%)*  **2) Antihypertensive Drug Treatment**  ***All***  6% (95% CI -2 to 13%)*  ***High Risk***  13% (95% CI 3 to 25%)*  **(3) Referral to Dietician**  ***All***  -3% (95% CI -10 to 4%)*  ***High Risk***  3% (95% CI -5 to 11%)* |
| Jacobsen et al. 2006  *To assess whether Framingham risk scores help physicians recommend statin prescriptions to patients at increased global CHD risk. To assess e the effects of risk assessment on other prevention measures.* | RCT  Academic General Medicine Clinics, US | 368 patients; 164 phyisicans | Patient | **Intervention:** Documentation of CHD risk on front of patient chart; box for physician to check action steps  **Control:** Documentation of consensus targets and strategies for CHD risk reduction on front of patient chart; box for physician to check action steps  Note: Education on CHD risk provided to both groups in 1 hour seminar | NA | (1) Proportion of high risk (>20%) patients prescribed statin therapy  (2) Proportion of moderate risk (10-19%) patients prescribed statin therapy  (3) Proportion of patients (any risk) prescribed diet, exercise, smoking cessation, aspirin, HTN therapy.  (4) Proportion of patients with documented CHD risk in notes | (1) **Statin Prescriptions, high risk group**: 38%  (2) **Statin Prescriptions, mod risk group**: 16%  (3) **Diet Prescription:** *Self Counseled* 27%  *Referred* 9%  **Exercise Prescription**: *Self Counseled* 12%  *Referred* 2%  **Smoking Cessation Prescription**:  *Self Counseled* 30%  *Referred* 0%  **Aspirin Prescription**: 11%  **HTN Prescription**: 12%  4) **Documentation of** **CHD risk in notes:** 65% | (1) **Statin Prescriptions, high risk group**: 40%  (2) **Statin Prescriptions, mod risk group**: 26%  (3) **Diet Prescription:** *Self Counseled* 16%  *Referred* 7%  **Exercise Prescription**: *Self Counseled* 9%  *Referred* 3%  **Smoking Cessation Prescription**:  *Self Counseled* 44%  *Referred* 13%  **Aspirin Prescription**: 14%  **HTN Prescription**: 14%  (4) **Documentation of** **CHD risk in notes:** 74% | (1) **Statin Prescriptions, high risk group**: +2%, p 0.86  (2) **Statin Prescriptions, mod risk group**: +10%, p0.18  (3) **Diet Prescription:** *Self Counseled -*11%, p 0.01  Referred-2%, p 0.55  **Exercise Prescription**: *Self Counseled* -3%, p 0.45  *Referred* +1%  **Smoking Cessation Prescription**:  *Self Counseled* +14%, p 0.11  *Referred* +13%, p 0.01  **Aspirin Prescription**: +3%, p 0.35  **HTN Prescription:** +2%, p0.72  (4) **Documentation of** **CHD risk in notes: +**9%, p 0.05 |
| Lowensteyn et al  1998  *To determine the feasibility of patient-specific computerized CHD risk profiles as clinical decision aids* | Cluster RCT  General practices, Canada | 958 patients, 253 physicians, unknown # practices | Physician | **Intervention:**  Computerized CHD risk profile for their patients after baseline  **Control:**  No profile | 3months | (1) Clinical follow-up in high (H) versus low (L) risk patients  (2) Change in CHD risk factors (e.g. cholesterol, BP, BMI, Smoking, CHD risk) | **(1) Clinical Follow-up, H/L risk patients**  RR 1.23 (95% CI 0.96 to 1.6)  **(2) Change in total cholesterol**  -0.09 mmol/L  **(2) Change in LDL cholesterol**  -0.01 mmol/L  **(2) Change in SBP**  -1.2 mmHg  **(2) Change in BMI**  -0.3 kg/m2  **(2) Change in smokers**  -2.3%  **(2) Change in CHD risk**  -0.3% | **(1) Clinical Follow-up,**  **H/L risk patients**  RR 0.77 (95% CI 0.58 to 1.03)  **(2) Change in total cholesterol**  -0.49 mmol/L  **(2) Change in LDL cholesterol**  -0.40 mmol/L  **(2) Change in SBP**  -2 mmHg  **(2) Change in BMI**  -0.2 kg/m2  **(2) Change in smokers**  -1.5%  **(2) Change in CHD risk**  -1.8% | **(1) Clinical Follow-up, H/L risk patients**  RR 0.46 (95% CI 0.08 to 0.87)  **(2) Change in total cholesterol**  -0.24 mmol/L†, p 0.05  **(2) Change in LDL cholesterol**  -0.23 mmol/L†, p 0.05  **(2) Change in SBP**  -0.8 mmHg†, p0.61  **(2) Change in BMI**  0.1 kg/m2†, p 0.31  **(2) Change in smokers**  0.8%†, p0.64  **(2) Change in CHD risk**  -1.4%†, <0.01 |
| Montgomery et al.  2000  *To investigate the effects of a computer-based decision support system + risk chart on absolute CVD risk, blood pressure, and prescribing of CVD drugs* | Cluster RCT  General practices, UK | 614 patients, 74 physicians/  11 nurses,  27 practices | Practice | **Intervention 1:**  Computer-based clinical decision support system + CHD risk chart  **Intervention 2:**  CHD risk chart alone  **Control:**  Usual care. | 12 months (for outcomes 1 and 2)  6 months (for outcome 3) | (1) 5-yr CHD risk ≥10%  (2) Blood pressure  (3) Prescribing of more than 1 CV drugs | **(1) CHD risk >10%**  88%  **(2) Systolic Blood pressure**  159 mmHg  **(2) Diastolic BP**  84 mmHg  **(3) Prescription of 2 CV drugs**  34%  **(3) Prescription of 3 or more CV drugs**  29% | **(1) CHD risk > 10%**  ***Computer +Chart***  89%  ***Chart alone***  85%  **(2) Systolic Blood pressure**  ***Computer +Chart***  153 mmHg  ***Chart alone***  153 mmHg  **(2) Diastolic BP**  ***Computer +Chart***  85 mmHg  ***Chart alone***  mmHg  86 mmHg  **(3) Prescription of 2 CV drugs**  ***Computer +Chart***  36%  ***Chart alone***  32%  **(3) Prescription of 3 or more CV drugs**  ***Computer +Chart***  25%  ***Chart alone***  35% | **(1) CHD risk > 10%**  ***Computer +Chart***  1%, adjusted‡ p 0.22  aOR‡ 1.7 (0.7 to 3.9)  ***Chart alone***  -3%. adjusted‡ p 0.43  aOR‡ 0.7 (0.3 to 1.6)  **(2) Systolic Blood pressure**  ***Computer +Chart***  5 mmHg, adjusted§ p NS  ***Chart alone***  4.6 mm Hg (95% CI, 0.8 to 8.4); adjusted§ p 0.02  **(2) Diastolic BP**  ***Computer +Chart***  1 mmHg, adjusted§ p NS  ***Chart alone***  2 mmHg, adjusted§ p NS  **(3) Prescription of 2 CV drugs**  ***Computer +Chart***  2%, p NR  ***Chart alone***  -2%, p NR  **(3) Prescription of 3 or more CV drugs**  ***Computer +Chart***  -4%, p NR  ***Chart alone***  +6%, p NR |
| Ramachandran et al.  2000  *To assess the appropriateness of lipid treatment decisions made by GPs* | Cross-sectional study (mailed postal question-naires)  UK | 61 physicians | NA | Lipid decisions in response to 20 patient case scenarios if CHD risk was calculated by physician (n=52%) or not (n=48%) | NA | (1) Proportion of correct responses to questions about the need for lipid medications|| | NR | NR | NR, p=0.21 |
| Van Steenkiste et. al. 2007.  To evaluate the effect of a CHD decision support tool on general practitioners (as regards clinical performance) and patients (as regards risk perception and self-reported lifestyle). | Cluster RCT  General Practices, Nether-lands | 39 practices; 45 General Practitioners; 623 patients. | Practice | **Intervention:** 4 hour interactive session on CHD risk and risk reduction for practitioners; 16 page decision support tools on CHD risk to be given to patient; 2 scheduled consultations to discuss risk  **Control**: Written educational materials for GPs on Dutch Choelsterol Guidelines | 26 weeks | (1) Physician Performance:  Appropriate ordering of cholesterol test  Appropriate smoking advice  Appropriate dietary advice  (2) Appropriate Patient Risk Perception, immediate  (3) Patient Lifestyle Changes:  Changes in Smoking, 26 weeks  Changes in Insufficient Physical Activity, 26 weeks | (1) **Physician Performance:**  **Appropriate ordering of cholesterol test:** 76% (62% to 86%)  **Appropriate smoking advice:** 91% (68 to 98%)  **Appropriate dietary advice:** 79% (58 to 91%)  (2) **Appropriate** **Patient Risk Perception, immediate:** 70%  **(3) Patient Lifestyle Changes**:  **Changes in Smoking, 26 weeks:** 0 %  **Changes in Insufficient Physical Activity, 26 weeks:** +4% | **(1) Physician Performance:**  **Appropriate ordering of cholesterol test:** 86% (75% to 92%)  **Appropriate smoking advice:** 82% (66% to 91%)  **Appropriate dietary advice:** 69% (55 to 81%)  **(2) Patient Risk Perception, immediate:** 72%  **3) Patient Lifestyle Changes**:  **Changes in Smoking, 26 weeks:** -4 %  **Changes in Insufficient Physical Activity, 26 weeks:** -7% | **(1) Physician Performance:**  **Appropriate ordering of cholesterol test: +**10%, NS  **Appropriate smoking advice:** -9%, NS  **Appropriate dietary advice:** -10%, NS  **(2) Patient Risk Perception, immediate:** +2%, NS  **(3) Patient Lifestyle Changes:**  **Changes in Smoking:** 4%, NS  **Changes in Insufficient Physical Activity, 26 weeks:** 11%, p<0.05 |

N reflects number of participants enrolled, which is not necessarily the same as the number who completed follow-up. RCT = randomized controlled trial; CV = cardiovascular; CHD = coronary heart disease

* Not reported in original paper, but calculated by systematic review team

†Mean difference and p-value adjusted for baseline differences using ANCOVA

‡ Adjusted for practice computer system and baseline CHD risk

§ Adjusted for practice computer system and baseline BP

|| Correct responses based on UK guidelines at the time (e.g. therapy indicated for those with a 10-year CHD risk ≥30%).
