# Supplementary material for: Does the routine use of global coronary heart disease risk scores translate into clinical benefits or harms? A systematic review of the literature
Source: BMC Health Serv Res. 2008 Mar 20;8:60. doi: 10.1186/1472-6963-8-60 (PMC2294118; doi:10.1186/1472-6963-8-60)
Supplement: Additional file 3 — Characteristics of studies addressing the harms of global risk scores, table summarizing study characteristics [file 1472-6963-8-60-S3.doc]

**Additional file 3: Characteristics of studies addressing the harms of global risk scores**

| **Study/**  **Research Objective** | **Design & Setting** | **Sample size** | **Unit**  **Of**  **Random-ization** | **Comparison** | **Duration**  **of Follow-up** | **Endpoints** | **Outcome** | | |
| --- | --- | --- | --- | --- | --- | --- | --- | --- | --- |
|  |  |  |  |  |  |  | **Control** | **Intervention** | **Difference** |
| Christensen, 1995.  *To investigate the psychological reactions of men diagnosed as having increased risk for ischemic heart disease* | Prospective Cohort  General practices in 2 municip-alities in Denmark. | 361 men | NA | Health screening with notification of increased risk status. Immediate counseling and 6 month f/u consultation.  Health screening with notification of low/moderate risk status. Immediate counseling, but no follow-up. | 6 months | (1) Change in General Health Questionnaire (GHQ)-12 Scores | (1) Change in GHQ 12 Score: -0.61 | (1) Change in GHQ 12 Score: -0.81 | (1) Change in GHQ 12 Score: -0.20, p 0.80 |
| Christensen, 2004  *To investigate the longterm psychological reactions to information about CHD risks.* | RCT  All 9 General Practices in 1 county in Denmark | 1,507 patients | Patients | Health screening with written notification of CHD risk and optional follow-up  Health screening with written notification about CHD risk and annual planned health discussion with GP  No screening or discussion | 12 months  and 5 years | (1) Change in GHQ-12 Scores | (1) Change in GHQ at 12 months  -0.16  (1) Change in GHQ at 5 years  -0.39 | (1) Change in GHQ at 12 months  0.05  (1) Change in GHQ at 5 years  -0.23 | (1) Change in GHQ at 12 months  +0.21, p 0.60  (1) Change in GHQ at 5 years  +0.16, p 0.73 |
| Connelly et al.  1998  *To identify the psychological effects of labeling men as having above average risk for CHD* | Cohort  General practices; UK | 5772 patients;  9 practices | NA | Mailed notification of higher than average risk status with immediately scheduled follow-up and offer to enter clinical trial*  Mailed notification of low risk status | 3 months | (1) Change in General Health Questionnaire (GHQ)-28 Score  (2) Transition from non-case to case status on GHQ (i.e. development of significant symptoms)  (3) Change in Speilberger state-trait anxiety inventory score | **(1) Change in General Health Score**  ***Low risk, 6 practices***  -0.23  ***Low risk, 3 practices***  -0.48  **(2) Transition to GHQ Case**  ***Low risk, 6 practices***  0.4%  ***Low risk, 3 practices***  0.3%  **3) Change in Anxiety Score**  ***Low risk, 6 practices***  -0.5  ***Low risk, 3 practices***  -1.2 | **(1) Change in General Health Score**  ***High risk, 6 practices***  -0.41  ***High risk*******, 3 practices***  -0.96  ***Moderate risk*******, 3 practices***  +0.22  **(2) Transition to GHQ Case**  ***High risk, 6 practices***  1.3%  ***High risk*******, 3 practices***  -4.8%  ***Moderate risk*******, 3 practices***  +4.6%  **(3) Change in Anxiety Score**  ***High risk, 6 practices***  -1.0  ***High risk*******, 3 practices***  -1.5  ***Moderate risk*******, 3 practices***  -0.6 | **(1) Change in General Health Score**  ***High risk, 6 practices***  -0.18, 95% CI - 0.93 to 0.57†  ***High risk*******, 3 practices***  -0,48, 95% CI -1.5 to +0.59†  ***Moderate risk*******, 3 practices***  +0.70, 95% CI -0.16 to 1.6†  **(2) Transition to GHQ Case**  ***High risk, 6 practices***  0.9%, p NR  aOR‡1.22 (0.81 to 1.83)  ***High risk*******, 3 practices***  -5.1%, p NR  aOR‡0.74 (0.36 to 1.51)  ***Moderate risk*******, 3 practices***  +4.3%, p NR  aOR‡1.80 (1.19 to 2.71)  **(3) Change in Anxiety Score**  ***High risk, 6 practices***  -0.5, 95% CI -1.7 to 0.66†  ***High risk*******, 3 practices***  -0.3, 95% CI -2.0 to 1.4†  ***Moderate risk*******, 3 practices***  +0.6, 95% CI -0.7 to 1.9† |
| Marteau et al.  1996  *To determine the whether a population-based intervention program to reduce CVD raises concerns about health or undermines a belief in the ability to reduce risk* | RCT  General practices; UK | 2984 married couples | Patient | Screening for and notification about CHD risk (with scheduled follow-up visit and counseling at intervals appropriate to risk).§  No screening or estimation of CHD risk | 12 months | (1) Perceptions of health  (2) Perceived risk of suffering a heart attack  (3) Perceived ability to reduce heart attack risk | (1) Perceived excellent/good health  75.7%  (2) Perceived lower than average risk of CHD  37.9%  (3) Perceived ability to reduce risk  81.8% | (1) Perceived excellent/good health  80.7%  (2) Perceived lower than average risk of CHD  42.1%  (3) Perceived ability to reduce risk  74.6% | (1) Perceived excellent/good health  +4/9%. P<0.001  (2) Perceived lower than average risk of CHD  3.9%, p 0.01  (3) Perceived ability to reduce risk  -6.4%, <0.001 |
| Meland, 1996.  *To study if an opportunistic screening of CHD risk factors among male attenders in general practice influenced subjective satisfaction with life of persons labeled high risk compared to other screened person* | Cross-sectional study  22 centers in Bergen, Norway. | 240 men | NA | Labelled high risk and invited for participation in RCT to promote risk reduction  Labelled low risk and not invited to participate | NA | (1) change in overall satisfaction with life question (OSwL) | (1) Change in OSwL: 0.3 | (1) Change in OSwL: 0.4 | (1) Change in OSwL: +0.1, p 0.9∞ |

RCT = randomized controlled trial; CV = cardiovascular; CHD = coronary heart disease

* 3 of nine practices used an intermediate risk notification in which they were told they were at moderate risk and encouraged to schedule follow-up; for analysis these moderate/high risk categories were collapsed.

† Not reported in original paper, but calculated by systematic review team

‡Adjusted for self described risk, perceived risk, , social class, life events, family history of CHD, BP

§ Follow-up intervals for top risk quintile every 2 months, fourth quintile every 3 months, third quintile every 4 months, second quintile every 6 months, and bottom quintile every year.

∞ Higher values correspond to higher dissatisfaction on 7 pt Likert scale
