# Supplementary material for: Does the routine use of global coronary heart disease risk scores translate into clinical benefits or harms? A systematic review of the literature
Source: BMC Health Serv Res. 2008 Mar 20;8:60. doi: 10.1186/1472-6963-8-60 (PMC2294118; doi:10.1186/1472-6963-8-60)
Supplement: Additional File 4 — Summary of overall study quality rating, table summarizing study quality ratings [file 1472-6963-8-60-S4.doc]

**Additional file 4: Summary of overall study quality ratings**

| **Study** | **Composite Quality Score** | **Overall Quality Grade** | **Quality Critique** |
| --- | --- | --- | --- |
| Hall et al, 2003. | 1.36* | Fair | - Alternate allocation of patients, rather than true randomization. - Minimal baseline patient or physician characteristics provided - Partially blinded assessment of outcomes - Did not account for clustering in analysis |
| Jacobsen et all, 2006 | 1.43† | Fair | - No information on baseline characteristics of physicians, but all medical residents - 5% false inclusions (differential) excluded from analysis - Didn’t account for multiple comparisons - Underpowered for primary and secondary analysis |
| Lowenstyn et al, 1998 | 0.79 | Methodologically limited | - Patient drop out rate at 3 months was 70% with no intention-to-treat analysis - Unequal measurement among intervention and control groups - No report of methods for outcome assessment |
| Montgomery et al, 2000. | 1.29 | Fair | - CHD risk scores applied in a mixed population both without and with CVD; tools not validated for use in secondary prevention population - No baseline physician characteristics provided - Non-blinded assessment of outcomes |
| Ramachandran et al, 2000. | 0.50 | Methodologically limited | - Response rate of only 31%. - No baseline physician characteristics provided and no control for potential confounders. - No assessment of ordering effects of case scenarios as potential bias in measurement. |
| Van Steenkiste, 2007 | 1.00 | Fair | - Non-blinded assessment of outcomes - Patient dropout ~30%, although reportedly no difference in non-responders - Didn’t account for multiple comparisons - Failed randomization; didn’t account for confounding in analysis |
| Christensen, 1995 | 1.14‡ | Fair | - No baseline characteristics presented, but would be expected to differ, and no control for potential confounding - CHD risk measurement not clearly valid or reliable - Patient dropout 23% |
| Christensen, 2004 | 1.21§ | Fair | - Patient drop-out 15-20% - CHD risk measurement not clearly valid or reliable |
| Connelly, 1998 | 0.57 | Methodologically limited | - No baseline characteristics of patients provided, but would be expected to differ - Risk levels not consistently defined - Known confounders were controlled for in analysis, but potential for unmeasured confounding - Patient dropout ~35% |
| Marteau, 1996 | 0.71∞ | Methodologically limited | - Limited baseline characteristics of patients provided, so unclear if randomization worked - No baseline measures of health status, perceived risk, or motivation in control group - 13% attrition from intervention group; unknown attrition from control group - Non-blinded assessment of outcomes |
| Meland, 1996 | 0.70|| | Methodologically limited | - No baseline characteristics of patient provided; authors report similarities in measurement, but not actual characteristics - Questionable validity of outcome measure - Non-blinded assessment of outcomes - No control of potential confounders |

* Increased from 1.1 to 1.4 due to personal communication with author indicating that randomization worked and there was no need to control for confounders

† Increased from 1.35 to 1.43 due to personal communication with author indicating sampling of all eligible patients, blinded assessment of outcomes, and attempted analysis accounting for clustering which showed no difference than the reported analysis without clustering

‡Increased from 1.07 to 1.14 due to personal communication with author indicating blinded assessment of outcomes

§ Increased from 1.14 to 1.21 due to personal communication with author indicating blinded assessment of outcomes

∞Increased from 0.57 to 0.71 due to personal communication with author, which included improved detail about the study intervention.

|| Increased from 0.50 to 0.70 due to personal communication with author, indicating validation work for the CHD risk measurement and satisfaction with life question.
